# Comparisons of predictors for typhoid and paratyphoid fever in Kolkata, India

**DOI:** 10.1186/1471-2458-7-289

**Published:** 2007-10-12

**Authors:** Dipika Sur, Mohammad Ali, Lorenz von Seidlein, Byomkesh Manna, Jacqueline L Deen, Camilo J Acosta, John D Clemens, Sujit K Bhattacharya

**Affiliations:** 1 National Institute of Cholera and Enteric Diseases, Kolkata, India; 2 International Vaccine Institute, Seoul, Korea; 3 London School of Hygiene and Tropical Medicine, London, UK

## Abstract

**Background:**

Exposure of the individual to contaminated food or water correlates closely with the risk for enteric fever. Since public health interventions such as water improvement or vaccination campaigns are implemented for groups of individuals we were interested whether risk factors not only for the individual but for households, neighbourhoods and larger areas can be recognised?

**Methods:**

We conducted a large enteric fever surveillance study and analyzed factors which correlate with enteric fever on an individual level and factors associated with high and low risk areas with enteric fever incidence. Individual level data were linked to a population based geographic information systems. Individual and household level variables were fitted in Generalized Estimating Equations (GEE) with the logit link function to take into account the likelihood that household factors correlated within household members.

**Results:**

Over a 12-month period 80 typhoid fever cases and 47 paratyphoid fever cases were detected among 56,946 residents in two *bustees *(slums) of Kolkata, India. The incidence of paratyphoid fever was lower (0.8/1000/year), and the mean age of paratyphoid patients was older (17.1 years) than for typhoid fever (incidence 1.4/1000/year, mean age 14.7 years). Residents in areas with a high risk for typhoid fever had lower literacy rates and economic status, bigger household size, and resided closer to waterbodies and study treatment centers than residents in low risk areas.

**Conclusion:**

There was a close correlation between the characteristics detected based on individual cases and characteristics associated with high incidence areas. Because the comparison of risk factors of populations living in high versus low risk areas is statistically very powerful this methodology holds promise to detect risk factors associated with diseases using geographic information systems.

## Background

Enteric fever is a systemic illness characterized by fever, abdominal pain, and non-specific symptoms including nausea, vomiting, headache, and anorexia. When enteric fever is caused by *Salmonella enterica *serovar Typhi, it is known as typhoid fever and when due to *S*. enterica serovar Paratyphi A, B, or C, it is called paratyphoid fever. The clinical differences in signs, symptoms and outcome between typhoid and paratyphoid fever are subtle [1]. Typhoid fever is traditionally believed to be more common, have a more severe clinical course, and result in more frequent and severe sequelae than paratyphoid fever. More recent studies suggest that paratyphoid fever has become the most frequent cause of enteric fever in some areas [2] and that the clinical presentation of typhoid and paratyphoid fever are impossible to distinguish [3] perhaps related to increasing use of antibiotics prior to presentation. Hospital-based studies and outbreak reports suggest that enteric fever is a major public health problem in India, with *S*. Typhi as the most common etiologic agent and an increasing proportion of cases due to *S*. Paratyphi A [4-8].

Risk factors for typhoid fever for the individual have been identified in several epidemiological studies suggesting either water-borne [9-13] or food-borne transmission [11,14-16]. A recent study by Vollaard, et al. [17] compared the risk factors for typhoid and paratyphoid fever in an urban slum of Jakarta, Indonesia and found a higher risk for transmission within the household for typhoid fever while paratyphoid fever seemed to be transmitted more frequently outside the household. These studies identified certain characteristics of the individual which may predict disease. The characteristics of the household, the neighbourhood and the wider area where the patient lives may influence the risk for diseases. Furthermore public health interventions such as water improvement or vaccination campaigns often have to target high risk areas as it is not feasible to supply interventions to extended areas and populations such as mega-cities. To make such interventions cost efficient it is essential to identify high risk population correctly.

In this paper, we compare and contrast risk factors of typhoid and paratyphoid fever and on an individual level as well for areas with increased incidence.

## Methods

### The study area

**Figure 1:**
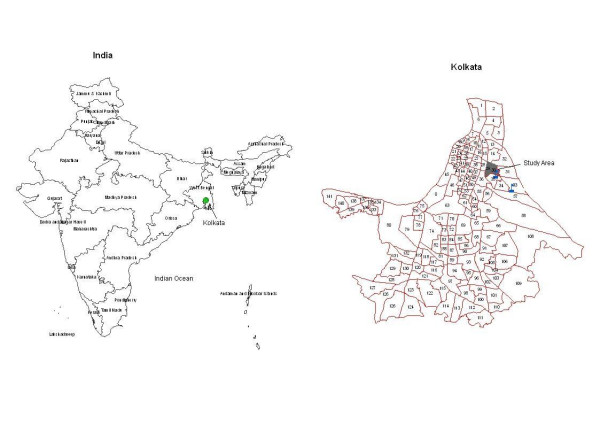
**The location of the study area in Kolkata, India**. The two referral hospitals of the typhoid program are shown in blue flags.

Kolkata is a densely populated mega-city with more than 13 million inhabitants, ranking among the top ten most populous cities of the world. About 40% of the population of Kolkata lives in *bustees*, officially designated slum areas. The city is divided into 141 administrative wards (Figure 1). The study site, Wards 29 and 30, was selected based on easy access from the research laboratory, the National Institute of Cholera and Enteric Diseases (NICED) and on the willingness of the community to participate in the study. The site is in eastern Kolkata, has a size of 0.99 km^2 ^and is representative for many slum areas in the city.

Water supply and sanitary facilities available to the population residing in the study sites are inadequate. Water is available through a limited number of municipal taps but only for several hours per day. The water and sewage pipelines lie close together and are prone to leakage and cross-contamination. The risk of contamination becomes particularly acute during the monsoon season when bustees are frequently flooded, and the water pressure outside the pipes exceeds the pressure inside the pipes. Common municipal latrines are shared by many families and sewage collects in open drains which overflow during the rainy season. Streets are narrow with huts and small shops encroaching onto the pavements. Uncovered food and cut fruits are sold by numerous open-air stalls.

### The population

A baseline census of the study population was carried-out from January to March 2003, followed by a second census one year later. During the baseline census, we recorded the age, sex, and educational level of each individual and collected household data including the demographics of household members, socio-economic status, water supply and sanitation [18]. The households had between 1 and 30 members (median 5), and occupied 1.5 rooms (mean). About 87% of the houses were *pucca *(made of bricks), 12% semi-*pucca *(of bricks combined with mud), and 1% *katcha *(of mud). During the census, each household and individual was assigned a unique identification number and household identification cards were distributed. Population movement between the first and second census was less than 5% suggesting a relatively stable population.

### The geographic information systems (GIS) database

We utilized IKONOS satellite imagery to construct the household geographic information systems (GIS) database of the study area [19]. In total, 10,954 households were registered in the baseline census. A ground survey was conducted to correlate each house parcel derived from the satellite imagery to a household census identification number. Two or more households sharing a single structure or closely connected structures were attached to a single parcel. During the ground survey, 10,954 households were correlated to 10,440 house parcels in the GIS database. Other than the households, the locations of project's health outposts, water bodies, roads and railway tracks were captured and recorded in the GIS database (Figure 1).

### The disease surveillance

**Figure 2:**
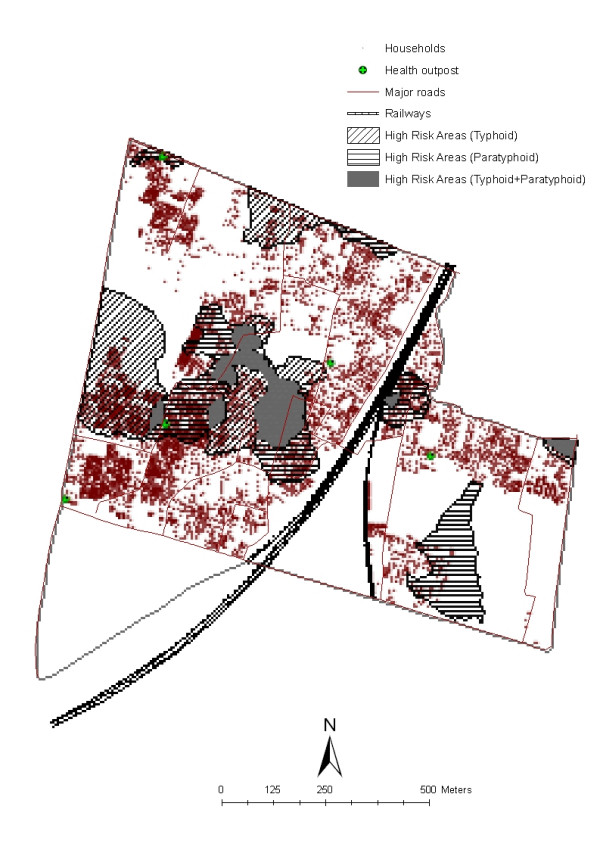
High risk areas of typhoid and paratyphoid fevers in the slums of Kolkata, India.

The study conducted disease surveillance through two project health outposts in referral hospitals, the B.C. Roy Children's Hospital and Infectious Diseases Hospital, as well as five community project health outposts (Figures 1 and 2). The hospital-based outposts were open 24 hours daily (3 shifts) whereas the community-based outposts were open from 8 am to 8 pm daily (2 shifts). The outposts offered free treatment for mild illnesses and blood culture for enteric fever. Community health workers visited each household in the study area once a month, inquired about fever cases, and encouraged them to visit project health outposts. Private practitioners in the area were visited regularly and encouraged to refer patients from the study area with fever to the outposts for diagnosis and treatment.

For each consenting (verbal) patient from the study population presenting with fever of 3 days or more, a case report form describing medical history, physical examination findings, and management was completed and blood was obtained. The patient's census ID from his/her household card was recorded on the case report form. In the absence of the household card, the census ID was retrieved from the computerized census database using the patient's name and other identifiers. Eight to ten millilitres of blood was collected from each adult participant and immediately inoculated into a Bactec plus F/Aerobic bottle (BD Bactec system, Franklin Lakes, NJ, USA). Five to seven millilitres of blood was collected from children less than 12 years of age and used to immediately inoculate a Bactec Peds Plus bottle (BD Bactec system, Franklin Lakes, NJ, USA). Laboratory-confirmed enteric fever cases were followed-up to inform patients about their diagnosis, verify identification, and record clinical outcome.

### Laboratory methods

The inoculated Bactec bottles were transported from the outposts to a laboratory at the National Institute of Cholera and Enteric Diseases three times per day. The bottles were incubated at 37°C for 7 days with subcultures on MacConkey agar (Difco, USA) during the first, second, fourth and seventh days of incubation. After every overnight incubation, non-lactose fermenting smooth colonies detected on the plates were checked for *Salmonella enterica *serotype typhi and *S. enterica *serotype *paratyphi *by Gram staining, and other biochemical tests following standard procedures [20]. Identification of *S*. Typhi was confirmed by slide and tube agglutination using polyvalent and monovalent factor antisera (Becton Dickinson, USA) for O and H antigen. Salmonella isolates were preserved in glycerol stock at -70°C and their identities confirmed at the University of Oxford-Welcome Trust Clinical Research Unit, Ho Chi Minh City, Vietnam.

### Data analysis

The presence or absence of characteristics of typhoid fever patients and paratyphoid fever patients was analyzed on two levels. In the first instance we compared the characteristics of individual typhoid fever patients, paratyphoid patients and people who had neither typhoid nor paratyphoid fever (table 1). In a second analysis we compared the aggregated characteristics of people living in high risk areas for acquiring typhoid fever, paratyphoid fever and areas with low risk for acquiring either infection (table 2). The definition of high and low risk areas is described below. The data defining the characteristics were extracted from the census database.

**Table 1:** Predictors of risk for typhoid and paratyphoid fever in the slums of Kolkata, India, 2003–2004

Variables	A	B	C	A vs. C	B vs. C	A vs. B
	
	Typhoid fever Cases (n = 80)	Paratyphoid fever Cases (n = 47)	Neither typhoid nor paratyphoid (n = 56,819)	OR* (P-value)	OR* (P-value)	OR* (P-value)
**Demographics**						

Age (mean, in years)	14.7	17.1	27.9	0.94 (<.01)	0.95 (<.01)	0.98 (.23)
Female	37 (46%)	19 (40%)	26,147 (46%)	0.97 (.91)	0.77 (.39)	1.25 (.54)
Illiterate	30 (38%)	12 (25%)	14,547 (26%)	1.28 (.30)	0.78 (.48)	1.74 (.17)
Percent household members under 15 years (mean, %)	35%	28%	29%	0.99 (.43)	0.98 (.04)	1.01 (.20)
Percent illiterate household members (mean, %)	35%	24%	27%	1.00 (.32)	0.99 (.20)	1.01 (.06)
Illiterate household head	29 (36%)	11 (23%)	16,117 (28%)	1.23 (.36)	0.68 (.27)	2.19 (.07)
Muslim individuals	46 (58%)	21 (45%)	23,749 (42%)	1.33 (.20)	0.84 (.55)	1.63 (.18)

**Socio-economic status**						

Renting accommodation	63 (79%)	29 (62%)	38,950 (69%)	1.44 (.18)	0.64 (.14)	2.26 (.04)
Household owning refrigerator	4 (5%)	3 (6%)	9,956 (18%)	0.28 (.01)	0.36 (.08)	0.74 (.71)
Household owning phone	4 (5%)	2 (4%)	8,745 (15%)	0.36 (.05)	0.29 (.08)	1.03 (.96)

**Water supply, sanitation, and hygiene**						

Household uses latrine for defecation	79 (99%)	46 (98%)	52,858 (93%)	5.32 (.09)	3.16 (.25)	1.78 (.68)
Always wash hands after defecation	47 (59%)	31 (66%)	38,227 (67%)	0.77 (.25)	1.02 (.92)	0.71 (.38)
Using tap water for drinking	76 (95%)	47 (100%)	55,641 (98%)	0.37 (.06)	--	--
Drinking water neither boiled nor filtered	77 (96%)	45 (96%)	52,392 (92%)	1.72 (.35)	1.57 (.53)	1.16 (.86)
No/rarely taken food from street vendor	39 (49%)	23 (49%)	22,783 (40%)	1.50 (.07)	1.50 (.16)	0.97 (.94)

**Location**						

Distance to water bodies from the household (meters)	152	149	152	0.99 (.35)	0.99 (.47)	1.00 (.90)
Population within a 50 meter radius	3065	2962	2560	1.00 (.25)	1.00 (.49)	1.00 (.88)

*OR, odds ratio adjusted for subject's age in a model using Generalized Estimating Equations (GEE) with the logit link function. The first variable "subject's age" is not adjusted

**Table 2:** Distribution of household level demographic and socio-economic characteristics (number and percentage in parentheses for the binary variables: 1–10, and average for the continuous variables: 11–18) in low and high risk areas for enteric fever

Variables	A	B	C	A vs. C	B vs. C	A vs. B
	
	TRA* (n = 1581)	PRA^† ^(n = 1487)	LRA^‡ ^(n = 6951)	OR (P-value)	OR (P-value)	OR (P-value)
**Demographics**						

Percent household members under 15 years (mean, %)	28%	24%	23%	5.32 (<.001)	1.12 (.09)	5.20 (<.001)
Percent illiterate household members (mean, %)	28%	25%	23%	3.00 (.001)	1.62 (.08)	1.31 (.22)
Illiterate household head	453 (29%)	399 (27%)	1675 (24%)	1.19 (.005)	1.14 (.05)	1.06 (.48)
Muslim households	1075 (68%)	528 (36%)	1861 (27%)	6.63 (<.001)	1.52 (<.001)	4.29 (<.001)

**Socio-economic status**						

Renting accommodation	1217 (77%)	1049 (71%)	4531 (65%)	1.67 (<.001)	1.26 (<.001)	1.31 (.001)
Household owning refrigerator	238 (15%)	269 (18%)	1108 (16%)	0.98 (.86)	1.19 (.02)	0.83 (.06)
Household owning phone	157 (10%)	208 (14%)	1063 (15%)	0.66 (<.001)	0.92 (.31)	0.72 (.005)

**Water supply, sanitation, and hygiene**						

Household using latrine for defecation	1362 (86%)	1453 (98%)	6573 (95%)	0.34 (<.001)	2.41 (<.001)	0.14 (<.001)
Always wash hands after defecation	871 (55%)	1105 (74%)	4851 (70%)	0.55 (<.001)	1.26 (<.001)	0.44 (<.001)
Using tap water for drinking	1457 (92%)	1445 (97%)	6901 (99%)	0.07 (<.001)	0.24 (<.001)	0.32 (<.001)
Drinking water neither boiled nor filtered	1464 (93%)	1364 (92%)	6384 (92%)	1.02 (.85)	0.96 (.70)	1.04 (.73)
Never/rarely consume food from street vendor	435 (28%)	898 (60%)	2765 (40%)	0.61 (<.001)	2.35 (<.001)	0.25 (<.001)

**Location**						

Distance to water bodies from the household (meters)	131	119	156	-30.21 (<.001)	-38.76 (<.001)	11.34 (<.001)
Population within a 50 meter radius	3158	2629	2052	961.83 (<.001)	541.52 (<.001)	406.93 (<.001)

*TRA = Typhoid high risk areas

^†^PRA = Paratyphoid high risk areas

^‡^LRA = Low risk areas of typhoid and paratyphoid

Age adjusted odds ratios with two-tailed p-values (given in parenthesis) were calculated for the cited binary variables (1–10) using logistic regression, and age adjusted parameter estimates with two-tailed p-values (given in parenthesis) were calculated for the cited continuous variables (11–18) using ordinary regression.

A total of 589 households living in the high risk areas of both typhoid and paratyphoid were removed from the analysis.

Five demographic characteristics were explored on the individual as well as on an aggregated level: household size (number of household members), the percentage of household members less than 15 years of age, and the percentage of illiterate household members were kept as a continuous variable. The educational attainment of the household head was categorized as literate/illiterate. Households were categorized as Muslim/non-Muslim (the majority of the population are Hindu or Muslim). In addition age, sex, and literacy of the individual are described in table 1.

The socioeconomic status of the household was explored through three characteristics: does the household rent the residence, does the household own a refrigerator, and does the household own a phone. Water supply, sanitation, and personal hygiene was characterized by five characteristics: whether or not the household members use a latrine, whether or not household members report to wash their hands always after defecation, whether or not household members use tap water for drinking, whether their drinking water was treated by boiling or filtering and drank untreated and finally whether respondents reported rarely or regularly to consume street food. The location of the household was assessed in two continuous variables: the distance to the nearest water body, which could be a pond, lake or river, and the population density measured as residents within a 50 meter radius. Distances to the nearest waterbodies inside the study area were measured as linear distance between geographically referenced points.

For the analysis by high risk area, the presence or absence of characteristics described for the individual and household level was re-analyzed for high and low risk areas for enteric fever. We used kriging [21,22], a geostatistical technique, to describe spatial patterns of typhoid and paratyphoid fevers in the study area. By use of kriging, disease incidences were extrapolated at regularly-spaced intervals that yielded disease surface maps which were classified into quintiles of predicted risk. Areas which fell into the highest quintiles of typhoid or paratyphoid fever incidence were defined as the high risk areas (Figure 2). Three areas were distinguished: high risk areas for typhoid fever, high risk areas for paratyphoid fever, and areas which did not fall in the highest quintile of either typhoid or paratyphoid risk (defined as low risk areas). A population of 2924 people residing in areas which were high-risk for both typhoid and paratyphoid fever was excluded from this risk area analysis.

Individual variables were fitted in Generalized Estimating Equations (GEE) with the logit link function to take into account the likelihood that household level factors correlated within household members [23]. We developed the models for both typhoid and paratyphoid fever. The models took diagnosis of typhoid or paratyphoid fever during the surveillance period (yes/no) for each analyzed individual as the dependent variable and fitted covariates as independent variables in the models. Coefficients of independent variables in the models were exponentiated to estimate the odds ratio of the disease risk. Standard errors for the coefficients were used to estimate p-values and associated 95% confidence intervals for the odds ratios. Statistical significance was designated as a p-value less than 0.01 as multiple hypotheses were tested. The statistical analyses were performed using Stata (Stata, Release 9.1, College Station Texas) software.

### Ethics

The study received approval from the Health Ministry Screening Committee of the Government of India and the Secretariat Committee for Research Involving Human Subjects, World Health Organization, Geneva, Switzerland.

## Results

Between 1 November 2003 and 30 October 2004 we detected 127 laboratory-confirmed enteric fever cases in a population of 56 946, of which 80 (63%) were due to typhoid fever and 47 (37%) paratyphoid fever. Note that 13% (21.2% typhoid, 25.5% paratyphoid) of the patients took antibiotics before coming to the health outpost. The incidence of paratyphoid fever was lower (0.8/1000/year), than the incidence of typhoid fever (incidence 1.4/1000/year).

### Age

Paratyphoid patients were older (mean age 17.1 years) than typhoid fever patients (mean age 14.7 years; p = 0.23; Table 1). Also the percentage of household members under 15 years of age (35%) was higher for typhoid fever cases than paratyphoid cases (28%) or people free of enteric fever (29%). The differences were not statistically significant on the individual level but highly significant differences were noted between risk areas. About 28% of household members in high risk areas for typhoid fever were less than 15 years of age, 24% in high risk areas for paratyphoid fever and 23% in areas with low risk for enteric fever (Table 2).

### Literacy

Literacy of typhoid patients was low (65%) compared to paratyphoid patients (76%) and people free of enteric (fever 73%, Table 1). Similarly the literacy of household-heads of typhoid fever patients (64%) was lower than for paratyphoid patients (77%) or the enteric fever free population (72%). The differences in literacy rates were not statistically significant on an individual level but the percentage of illiterate household members and household heads (28% and 29%) was significantly higher in the high risk areas for typhoid fever compared to low risk areas for enteric fever (23% and 24%, Table 2).

### Religion

Typhoid cases were more frequently Muslims (58%) than paratyphoid cases (45%) or people free of enteric fever (42%, Table 1). Similarly the percentage of Muslim households was significantly higher in high risk areas for typhoid fever (68%) than in high risk areas for paratyphoid fever (36%) or low risk areas for typhoid fever (27%, Table 2).

### Socio-economic status

Only 15% of the households in the study area owned a telephone, 18% owned a refrigerator, and 69% had to rent accommodation in contrast to owning their accommodation. Typhoid fever cases were more likely to live in rented accommodations (79%) and less likely to own a refrigerator (5%) or a mobile phone (5%) compared to the population free of enteric fever (69%,18%, 15% respectively, Table 1). The percentage of residents in high risk areas for typhoid fever renting accommodation (77%) was significantly higher than in high risk areas for paratyphoid fever (71%) or low risk areas for enteric fever (65%, Table 2). Similarly telephone ownership was significantly lower in high risk areas for typhoid fever (10%) compared to high risk areas for paratyphoid fever (14%; p = 0.005) or low risk areas for enteric fever (15%; p < 0.001).

### Water supply, sanitation, and hygiene

The large majority of the population in the study site (93%) used latrines for defecation. The percentage of latrine users was even higher among typhoid patients (99%) and paratyphoid patients (98%; Table 1). Paradoxically latrine use was lower (86%) in the high risk areas for typhoid fever than in high risk areas for paratyphoid fever (98%) or low risk areas for enteric fever (95%). Among typhoid fever cases 59% stated that they always washed their hands after defecation in contrast to 67% of the general population (p = 0.25) and 66% of paratyphoid cases (p = 0.38). In low risk areas for enteric fever 70% of respondents stated they always wash their hands after defecation, in contrast to 74% in high risk areas for paratyphoid fever (p < 0.001) and only 55% in high risk areas for typhoid fever (p < 0.001).

All paratyphoid fever cases and 98% of people free of enteric fever stated that they use tap water for drinking. Among typhoid fever cases the percentage was 95% suggesting that 5% obtain drinking water from other sources such as ponds. Also typhoid fever cases (96%) were more likely to drink water without boiling or filtering compared to 92% people free of enteric fever. In high risk areas for typhoid fever use of tap water was significantly lower (92%) compared to high risk areas for paratyphoid fever (97%) or low risk areas for enteric fever (99%). In contrast there was no significant difference in the treatment of drinking water between the three risk areas.

Consumption of food from street vendors was higher among people free of enteric fever (60%) compared to typhoid (51%) and paratyphoid cases (51%). Even though in high risk areas for typhoid fever 72% of respondents suggested to consume such foods in contrast to 60% of respondents in low risk areas (p < 0.001).

### Location

The study area is a congested slum with an average of 2560 people residing within a radius of 50 meters. The density was higher for typhoid fever cases who had 3065 people/50 meter radius. The population density was highest in high risk areas for typhoid fever (3158 people/50 m radius), followed by high risk areas for paratyphoid fever (2629 people/50 m radius; p < 0.001), and lowest in low risk areas (2052 people/50 m radius; p < 0.001). Distance to waterbodies did not appear to correlate with risk for enteric fever. However high risk areas for paratyphoid fever tended to be close to water bodies (119 meters) than high risk areas for typhoid fever (131 meters) or low risk areas (156 meters; all comparisons p < 0.001).

## Discussion

The study explored risk factors for typhoid fever and paratyphoid fever and found several interesting trends such as a lower socio economic status amongst enteric fever cases compared to people free of enteric fever was observed. But besides young age, a well known risk factor for enteric fever, none of the characteristics reached the predetermined significance level (p < 0.01). Based on an analysis of individual risk factors it is not possible to distinguish whether the observed trends are due to chance or a small sample size. A secondary analysis found that trends observed between cases and population free of enteric fever was highly significant when the populations living in high risk areas and low risk areas were compared.

### Characteristics

A higher percentage of Muslims was also noted among typhoid fever cases than among people free of enteric fever on an individual basis. However this apparent higher risk for Muslims may be confounded by a higher percentage of children at risk for typhoid fever in Muslim communities than in Hindu communities. The higher percentage of Muslims among enteric fever patients on a household level was not statistically significant after adjusting for age. Differences in age alone did not account for the higher percentage of Muslims in the high risk areas, other factors, such as socioeconomic status, health education, hygiene, alone or in combination may be responsible for the overrepresentation of Muslims in high risk areas.

Illiteracy rates were found highest in the high risk areas for typhoid fever, followed by high risk areas for paratyphoid fever and were lowest in low risk areas for enteric fever. Younger people may have not yet had a chance to acquire education. To correct this potential confounding factor odds ratios were adjusted for age and remained highly significant. Furthermore we compared illiteracy in household heads, who are frequently the decision makers in health related aspects of the household. Illiteracy was highest among the household heads living in high risk areas for typhoid fever.

The socioeconomic influence on disease risk was assessed in three characteristics, ownership of the house, and ownership of two consumer goods: refrigerator and phone. The study found that in high risk areas for typhoid fever a significantly higher percentage of the population rented their accommodation compared to low risk areas or high risk areas for paratyphoid fever where ownership of premises by the residents is more frequent. Ownership of the refrigerator and phone was lowest among residents in the high risk areas for typhoid fever further suggesting a lower economic status in high risk areas for typhoid fever compared to high risk areas for paratyphoid fever and low risk areas. Other markers for economic status such as income are unlikely to be reliably reported during a brief interview at the time of census, are therefore unlikely to reflect the actual prosperity of the household. The study findings suggest that even within a slum population the economically most disadvantaged segment of the population is at highest risk for diseases of poverty such as enteric fever.

The absence of safe water supply and sanitation are believed to contribute to the transmission of enteric fever. The study found significantly lower latrine use in high risk areas for typhoid fever compared to low risk areas or high risk areas for paratyphoid fever. The study found no significant differences in the treatment of drinking water. In all areas the large majority of residents drink water which is neither boiled nor filtered. However tap water use was significantly lower in high risk areas for typhoid fever, intermediate in the high risk areas for paratyphoid fever and lowest in the low risk areas for enteric fever. This suggests that the source of drinking water correlates closer with the risk for enteric fever than the treatment of drinking water. Poor food hygiene is a risk factor for enteric fever. Food prepared and sold on the streets of resource poor countries are particularly hazardous as water and soap to clean plates and utensils are frequently missing and tropical climates can transform a food stall in an incubator. However in our study the enteric fever cases were more likely to state that they had never or rarely consumed street food compared to people free of enteric fever. Street food is more expensive than food prepared at home. It is possible that the purchase of street food is not affordable to the poorest segment of the study population and is therefore rather a marker of elevated socio economic status rather than a risk factor for enteric diseases.

Two geographic factors were studied, the distance to a body of water and population density. The presence of open water bodies, such as a pond, could contribute to the transmission of enteric fever in a variety of ways. The proximity of an open water body could predispose to use this water for washing and food preparation, activities which could transmit enteric fever. However the precise role of open water bodies was beyond the scope of this study and may also require more detailed analysis and additional study.

Population density, expressed here as people per 50 meter radius, was highest around typhoid fever cases, followed by paratyphoid fever cases and lowest around people free of enteric fever, though these differences were not statistically significant. The population density was significantly higher in high risk areas for typhoid fever than in low risk areas or high risk areas for paratyphoid fever suggesting that high risk areas for typhoid fever were most crowded. Crowding directly contributes to higher enteric fever rates by facilitating person to person transmission. Furthermore crowding may be a sign of depressed socioeconomic circumstances as people tend to escape crowded neighbourhoods if they can afford to move.

### Potential limitations

The comparisons between individuals with paratyphoid fever and typhoid fever are based on a limited number of cases (80 typhoid fever cases and 47 paratyphoid fever cases). While these are a large group of cases for a population based study the study may be underpowered to detect ecologic differences on an individual level. Secondly the associations detected in our study are data derived. It would be of interest to repeat the analysis with data collected in the future to see whether the high risk areas are permanent or change over time. Thirdly the data were collected in two low income slums. A similar study in economically more mixed areas of Kolkata could help further understanding of factors causally related to enteric fever. Finally the responses were elicited at the time of the census prior to the disease, which was detected in the 12 months period following the census. The questions did not address specific risk factors for an individual episode of enteric fever. However the prospective design of the study excludes the possibility that the event, enteric fever, can bias responses a potential problem in retrospective studies.

## Conclusion

Our findings suggest that enteric fever, thought to transcend social classes was inversely correlated with socio economic status in a slum of Kolkata. The subpopulation with the lowest socio economic status of this impoverished population appears at highest risk for enteric fever. The comparison between characteristics of typhoid fever and paratyphoid fever patients suggests differences in socio economic status, with a more depressed status among paratyphoid patients. Exploring characteristics not only on an individual level but also on a larger population level, i.e. between risk areas adds statistical power needed to detect subtle differences.

## Competing interests

The author(s) declare that they have no competing interests.

## Authors' contributions

MA and LVS contributed to the design and analysis of the study and the writing of the paper; DS, CJA, BM, and JLD contributed to the design of the study, implemented the execution and supervision of the study, and writing of the paper; JDC and SKB supervised the execution, and analysis of the study and contributed to the writing of the paper. All authors have read and approved the final version of the manuscript.

## Pre-publication history

The pre-publication history for this paper can be accessed here:


